# A Prospective Study on the Feasibility and Effect of an Optimized Perioperative Care Protocol in Pediatric Neuromuscular Scoliosis Surgery

**DOI:** 10.3390/jcm13247848

**Published:** 2024-12-23

**Authors:** Marie Mostue Naume, Christina Engel Hoei-Hansen, Alfred Peter Born, Ghita Brekke, Astrid Høj, Maja Risager Nielsen, Lise Borgwardt, John Vissing, Jesper Dirks, Anne Kathrine Stæhr Rye, Morten Hylander Møller, Thomas Borbjerg Andersen, Mette Cathrine Ørngreen

**Affiliations:** 1 Department of Pediatric and Adolescent Medicine, Copenhagen University Hospital, Rigshospitalet, 2100 Copenhagen, Denmark; marie.mostue.naume.01@regionh.dk (M.M.N.); alfred.peter.born@regionh.dk (A.P.B.); ghita.brekke@regionh.dk (G.B.); mette.cathrine.oerngreen.01@regionh.dk (M.C.Ø.); 2 Copenhagen Neuromuscular Center, Department of Neurology, Copenhagen University Hospital, Rigshospitalet, 2100 Copenhagen, Denmarkmajanielsen06@hotmail.com (M.R.N.); john.vissing@regionh.dk (J.V.); 3 Department of Clinical Medicine, University of Copenhagen, 2200 Copenhagen, Denmark; anne.kathrine.staehr.rye@regionh.dk (A.K.S.R.); morten.hylander.moeller@regionh.dk (M.H.M.); 4 Department of Clinical Physiology, Nuclear Medicine & PET, Copenhagen University Hospital, Rigshospitalet, 2100 Copenhagen, Denmark; lise.borgwardt@regionh.dk; 5 Department of Anesthesiology, Copenhagen University Hospital, Rigshospitalet, 2100 Copenhagen, Denmark; jesper.dirks@regionh.dk; 6 Respiratory Center East, Department of Anaesthesia, Pain, and Respiratory Support, Rigshospitalet Glostrup, 2600 Glostrup, Denmark; 7 Department of Intensive Care, Copenhagen University Hospital, Rigshospitalet, 2100 Copenhagen, Denmark; 8 Spine Section, Department of Orthopaedic Surgery, Copenhagen University Hospital, Rigshospitalet, 2100 Copenhagen, Denmark; thomas.borbjerg.andersen@regionh.dk

**Keywords:** neuromuscular scoliosis, optimized perioperative care protocol, pneumonia, nutrition, respiration

## Abstract

**Background/Objectives**: A recent retrospective study conducted by our team identified a high percentage of postoperative pneumonia in children with neuromuscular scoliosis. Based on the findings in that study and our clinical experience, we aimed to assess the effectiveness of an optimized perioperative care protocol. **Methods**: As part of a prospective study, a multidisciplinary team developed a protocol that included preoperative nutritional and respiratory optimization, intra- and postoperative intravenous glucose infusion, early extubation, and postoperative nutritional optimization. Non-ambulant children between 6 and 18 years of age with neuromuscular scoliosis were eligible for inclusion in the study. The primary outcome was the rate of postoperative pneumonia within 30 days of surgery. The secondary outcome measures were the rate of postoperative complications, including readmissions. All the outcomes were compared to a retrospective control group that was receiving standard care during the same period. **Results**: Eleven children were included in the intervention group and 14 in the control group. In regard to the intervention group, the nutritional and respiratory assessment before surgery resulted in optimized treatment in 8/11 patients (73%) and 9/11 patients (82%), respectively. One patient (9%) in the intervention group and three patients (21%) in the control group developed postoperative pneumonia (relative risk 0.42, 95% confidence interval 0.05–3.50). The intervention and control groups did not differ significantly in terms of postoperative complications or readmission rates. **Conclusions**: The multidisciplinary care protocol is feasible, with a high compliance rate in regard to study procedures. A numerical reduction in the 30-day pneumonia rate did occur in the intervention group; however, this reduction did not reach statistical significance.

## 1. Introduction

Children with neuromuscular scoliosis represent a heterogeneous group of patients with various neuromuscular disorders (NMDs) and cerebral palsy (CP). This patient group is prone to progressive scoliosis development and may need scoliosis surgery to relieve pain, to stabilize respiratory function, and improve sitting comfort [1,2]. However, there is a high risk of postoperative complications, such as acute respiratory failure, pneumonia, and wound infections, among others [3,4,5]. Studies have shown that these complications are associated with the patient’s preoperative nutritional status and decreased respiratory function, which in turn increase the risk of postoperative complications [3,6,7].

Reduced respiratory function, poor nutritional status, and low skeletal muscle mass due to immobility and muscular wasting are common in children with neuromuscular scoliosis [8,9,10,11]. Patients with low skeletal muscle mass are at increased risk of hypoglycemia during episodes of enhanced energy demand, such as during periods of fasting [12,13]. We have experienced children with low muscle mass developing metabolic complications postoperatively and their stay in the intensive care unit (ICU) is prolonged beyond what is expected. Based on our clinical experience, we conducted a retrospective study, investigating the five-year mortality and morbidity rate after scoliosis surgery in pediatric neuromuscular scoliosis patients [9]. We found that 13% of the children developed postoperative pneumonia [9]. Furthermore, another study of 111 pediatric patients with neuromuscular scoliosis, by Luhmann et al., identified a history of pneumonia and the presence of a gastrostomy tube as risk factors for developing pulmonary complications [14]. Similarly, Lee et al. found that pre-existing pulmonary disease, obesity, and cachexia predicted 90-day readmission in 2856 children with CP undergoing scoliosis surgery [3]. Rajkumar et al. found co-occurring postoperative complications in 11% of patients with neuromuscular scoliosis undergoing scoliosis surgery. Clinical factors such as impaired cognitive status, seizures, the need for preoperative nutritional support, and structural pulmonary abnormalities, increased the risk of these complications [15]. A recent study by Brusa et al., investigating secondary outcomes in patients with spinal muscular atrophy (SMA), found that scoliosis surgery had a positive effect on respiratory function. However, 7/10 patients reported postsurgical pain, reduced mobility, and unmet care needs [16]. Thus, the authors highlight the importance of multidisciplinary approaches and proactive initiatives to improve postoperative management in this patient group [16].

It has been suggested that the preoperative optimization of nutrition and respiratory care may decrease the length of stay in hospital and the risk of infection in pediatric patients with neuromuscular scoliosis [17,18]. Hence, optimizing the patient’s preoperative nutrition and respiratory status may reduce complications and readmissions [18]. Currently, there is a lack of prospective studies investigating the effect of preoperative nutritional and respiratory optimization in children with neuromuscular scoliosis undergoing scoliosis surgery. Developing and implementing effective optimization pathways for these patients is crucial to ensure the best possible outcome of surgery. We found in the previous retrospective study that children who developed postoperative pneumonia experienced a longer stay in the ICU and hospital [9]. This places a burden on the children, their relatives, and the healthcare system.

Based on our previous retrospective study and the published literature, we wanted to assess the effect of an optimized perioperative care protocol to reduce the risk of complications at our surgical site. We hypothesized that using an optimized perioperative care protocol would decrease the rate of pneumonia and complications after scoliosis surgery.

## 2. Materials and Methods

This was a prospective, single-site study. The study site was a tertiary center that serves approximately half of the country’s population (around three million people). The manuscript has been prepared according to the CONSORT statement [19].

### 2.1. Participants and Setting

This study was designed using a multidisciplinary approach to perioperative optimization in collaboration with anesthesiologists, intensive care specialists, orthopedic surgeons, neuropediatricians, dietitians, and pulmonary specialists. Eligible patients were non-ambulant children and adolescents between 6 and 18 years of age, who were scheduled for scoliosis surgery a minimum of three months after inclusion, with one of the following diagnoses: SMA, Duchenne muscular dystrophy (DMD), CP, merosin-deficient congenital muscular dystrophy (MDC1A), or Ulrich muscular dystrophy (UMD). The recruitment period was from March 2020 to March 2023. Eligible patients were enrolled by their treating pediatrician or orthopedic surgeon in the outpatient clinic.

### 2.2. Trial Protocol

**Figure 1:**
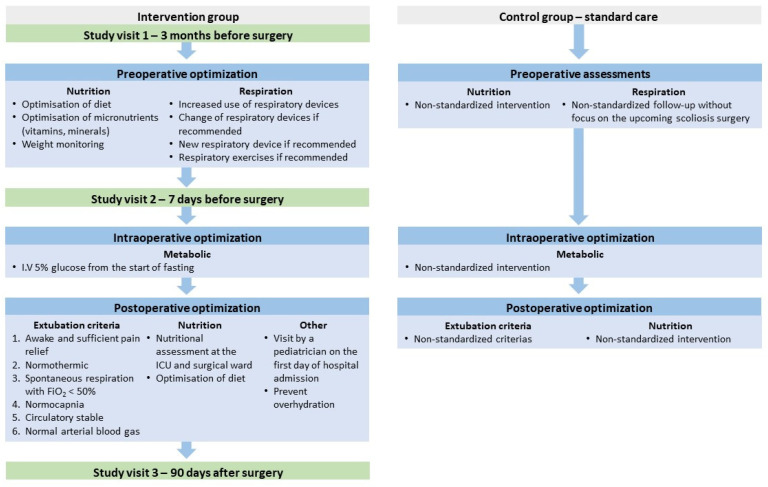
The perioperative optimization care protocol compared to the standard care. Abbreviations: FiO_2_ = fraction of inspired oxygen, ICU = intensive care unit, IV = intravenous.

The trial protocol included three hospital visits. A preoperative visit, a minimum of two months before surgery, a preoperative visit seven days before surgery, and a postoperative visit 90 days after surgery (Figure 1). The same assessments were conducted at each study visit. The study visits consisted of nutritional screening by a clinical dietitian, the patient’s parents completed a three-day food record, an indirect calorimetry test was performed to measure the daily resting energy expenditure (REE), and the child underwent a dual-energy X-ray absorptiometry (DXA) scan to assess their body composition and bone mineral density (BMD). Moreover, if the child was able to cooperate, they performed a lung function test (spirometry). The lung function test was only performed at visit 1. Blood samples were collected, measuring organ-specific markers, vitamins, and minerals. Lastly, the children and their parents completed quality of life (QOL) questionnaires during all the visits, both preoperatively and 90 days after surgery. A detailed description of the assessments during the study visits is provided in Protocol S1.

The perioperative care protocol included a preoperative nutritional and respiratory optimization plan (Figure 1). The nutritional optimization was based on nutritional screening, an indirect calorimetry test, food records, nutritional blood samples, and DXA scans to establish whether the participant was a candidate for nutritional optimization. The parameters were assessed at the three study visits. The nutritional screening of children with a neurological impairment using the European Society for Paediatric Gastroenterology, Hepatology and Nutrition (ESPGHAN) guidelines included one or more of the following red flag warning signs for the identification of undernutrition [20]: (1) physical signs of undernutrition, such as decubitus skin problems and poor peripheral circulation; (2) a weight-for-age z-score < −2; (3) a triceps skinfold thickness <10th percentile for their age and sex; (4) a mid-upper arm fat or muscle area <10th percentile; or (5) faltering weight and/or a failure to thrive.

Increasing the patient’s energy and/or protein intake was conducted by adding foodstuffs or initiating/increasing the use of oral nutritional supplements or tube feeding. The aim was to gradually increase the patient’s energy intake depending on their tolerance to the food, which was often challenging for those participants suffering from nausea, vomiting, reduced gastric emptying, and constipation. Weight monitoring was conducted at each study visit and every four weeks (phone or e-mail consultation) between visits to guide energy optimization. Increased micronutrient intake was conducted through supplementation, but could also be increased through possible oral nutritional supplements or tube feeding. If the patient was not found to be at nutritional risk according to any of the red flag warning signs, optimization could still be deemed relevant according to the results of the food records, nutritional blood samples, and DXA scans (see Table S1). One patient was deemed to be overweight, according to the clinician’s perspective, hence the energy intake was intentionally calculated to be below the measured energy needs.

Before the study, the standard care for patients with neuromuscular diseases, such as SMA and DMD, was to refer the patient to the highly specialized respiratory centre, Respiratory Centre East (RCE), if they were wheelchair dependent, had a forced vital capacity <80% of what was expected, or if they had experienced recurrent pneumonia. However, there were no standard guidelines for the referral to RCE for patients with CP. Furthermore, for those patients already monitored by RCE, there were no specific guidelines on a standardized intervention or follow-up at RCE before scoliosis surgery. The optimization of the respiratory function included a consultation at RCE. The pulmonary specialists decided whether secretion management treatment, including intermittent continuous positive airway pressure (CPAP) therapy, mechanical insufflation-exsufflation (MI-E) therapy, or isotonic saline nebulization should be started or intensified. They also decided whether a test for a sleep-related breathing disorder should be performed and whether a CPAP treatment, for nightly use, should be started or optimized. The intraoperative optimization included infusion with a 5% glucose solution to prevent hypoglycemia. The postoperative optimization included early extubation with new extubation criteria, nutritional screening, and a consultation with a pediatrician (Figure 1).

### 2.3. Outcome Measures

#### 2.3.1. Primary Outcome Measure

The primary outcome was the postoperative pneumonia rate within 30 days of surgery. Pneumonia was diagnosed by a medical doctor and based on clinical features, such as cough, fever, and lung imaging.

#### 2.3.2. Secondary Outcome Measures

1. The duration of mechanical ventilation in hours from the end of surgery to extubation;
2. The days admitted to the ICU;
3. The rate of bacteremia within 30 days of surgery;
4. Wound infections within 30 days of surgery;
5. The duration of the stay in hospital;
6. Reoperation rates (a surgical procedure related to the primary surgical procedure) within 30 days of surgery;
7. Readmission rates within 30 days of surgery.

In the intervention group, the following outcomes were also registered:

8. The effect of preoperative optimization from visit 1 to visit 2 in terms of the patient’s nutritional status, QOL, body composition, and BMD measured by DXA;
9. A postoperative assessment 90 days after surgery: an assessment of patient’s REE, weight, nutritional status, body composition, and BMD measured by DXA and the patient’s QOL.

### 2.4. Control Group

The intervention group was compared with a concurrent control group of patients with neuromuscular scoliosis who underwent scoliosis surgery at the same surgical site, receiving standard care. The control group consisted of children who did not participate in the study because their preoperative optimization period was less than two months, as well as those who were not invited to join the intervention group. For example, some CP patients were not identified as eligible candidates within the time frame because they attended other hospitals and were referred to the site for surgery. To ensure comparability with the patients in the intervention group the inclusion criteria for the control group were identical to those in the intervention group, namely non-ambulant children and adolescents between 6 and 18 years of age, with one of the following diagnoses, SMA, DMD, CP, MDC1A or UMD, who underwent scoliosis surgery from March 2020 to March 2023. The control group data were retrieved from the patient’s medical files retrospectively. An approval from the Data Protection Agency (P-2020-51) and the team for medical data (J-23072196) was conducted to assess the retrospective data in the control group.

### 2.5. Sample Size

Approximately one child with neuromuscular scoliosis undergoes scoliosis surgery every month at the trial site. We aimed to include as many eligible children as possible within the recruitment period. Thus, no sample size calculation was made.

### 2.6. Statistical Analysis

The statistical program R version 4.2.0, with R studio version 2022.02.2 (R Foundation for Statistical Computing, Vienna, Austria), was used to perform the statistical analyses. Continuous data are presented as the median with a range. Categorical data are presented as numbers and percentages. We compared the continuous data for the patients by using the non-parametric Wilcoxon test and the Kruskal–Wallis test. Categorical data were compared by using Fisher’s exact test. A *p*-value < 0.05 was considered statistically significant. We calculated the relative risk and 95% confidence intervals (CIs) between the intervention and the control group for the primary outcome. We refrained from performing correlation and association analysis due to the low number of patients in the study.

## 3. Results

There were 16 patients who were eligible to participate in the study, of whom 14 accepted. Three of the patients were excluded because their surgery was postponed. The five patients who did not participate or were excluded had CP (*n* = 3, 60%) and SMA (*n* = 2, 40%). The median age in that group was 13 years (range, 8–17). The results from the 11 patients in the intervention group were compared to the 14 patients in the control group.

### 3.1. Compliance with the Trial Protocol

The median preoperative optimization period between visits 1 and 2 was 133 days, ranging from 67 to 562 days. All the patients in the intervention group completed QOL questionnaires.

#### 3.1.1. Respiratory Optimization

Eight of the 11 patients (73%) in the intervention group were already being monitored at RCE as part of the standard care. For those patients, a visit was secured and scheduled preoperatively. The three remaining patients had CP and were referred to RCE due to a history of respiratory challenges, since they were not able to perform a spirometry test. Thus, none of the patients performed a spirometry test. Nine out of 14 patients (64%) in the control group were being monitored at RCE as part of the standard care. The patient’s in the control group had a consultation at RCE a median of 132 days (26–1806 days) before surgery. A total of 82% (*n* = 9) of the patients in the intervention group were advised to use additional or other respiratory devices or to use the respiratory devices more intensively before surgery (Table 1). The three CP patients who were not being monitored at RCE before their inclusion in the study were advised to use an intermittent CPAP machine and MI-E (Table 1). Postoperatively, the early extubation criteria were used for all the patients in the intervention group. Thus, three patients (27%) were extubated in the operating room (OR) and all were extubated within the first 24 h after surgery. In the control group, five patients (36%) were extubated in the OR and all the patients were extubated within 24 h after surgery.

#### 3.1.2. Nutritional Optimization

We found that six patients (55%) in the intervention group were at nutritional risk at visit 1. Furthermore, the dietitian considered that eight (73%) patients needed nutritional optimization before surgery. Nutritional optimization consisted of changes in the macronutrient composition of their diet or adding dietary or nutritional supplements (Table S2). The REE was measured in seven (63%) patients, the rest of the patients were not able to cooperate. The average measurement duration was 9 min (range, 3–29), whereas the optimal measurement duration is 30 min. The REE and nutritional risk assessments were not conducted in the control group prior to surgery, as these assessments are not part of the standard care. The compliance in terms of the patient’s food record was 100% for visit 1, but decreased to 64% and 55% at visits 2 and 3. No food records were identified in the medical files of the patients in the control group. DXA scans were conducted for all the patients (100%) at visit 1 of the intervention group and decreased to 82% at visits 2 and 3. Whereas, three of the patients (21%) in the control group were DXA scanned prior to surgery. All patients in the intervention group were seen by a dietitian postoperatively. None of the patients in the control group were seen by a dietitian during hospitalization.

### 3.2. Demographics and Baseline Data

Seven of the 11 patients (64%) in the intervention group and 10 out of the 14 patients (71%) in the control group were partly or entirely fed by a gastrostomy tube. The nutritional status based on the patient’s weight-for-age z-score (WAZ) revealed that 4/11 (36%) and 1/11 (9%) of the patients in the intervention group were severely underweight or underweight, respectively. A high percentage of severely underweight and underweight patients was also present in the control group (Table 2). The median estimated energy need in the intervention group at visit 1 was 1346 kcal (1120–1792 kcal) compared to 1155 kcal (984–1884 kcal) in the control group. All the patients in both groups underwent posterior fusion surgery.

### 3.3. Outcomes

#### 3.3.1. Primary Outcome

One out of 11 patients (9%) in the intervention group and three out of 14 patients (21%) in the control group (*p* = 0.6, odds ratio 0.38, 95% CI of 0.006–5.7) developed postoperative pneumonia. The relative risk in the intervention group was 0.42, with a 95% CI of 0.05–3.5. The patient that developed pneumonia in the intervention group had MDC1A and the patient was diagnosed 21 days after discharge. The patient had a history of recurrent pneumonia and used assistive respiratory devices (NIV and intermittent CPAP) prior to visit 1. Of the three patients in the control group that developed pneumonia, one had MDC1A and two had CP. The CP patients were not monitored by RCE and did not use any respiratory devices.

#### 3.3.2. Secondary Outcomes

**Figure 2:**
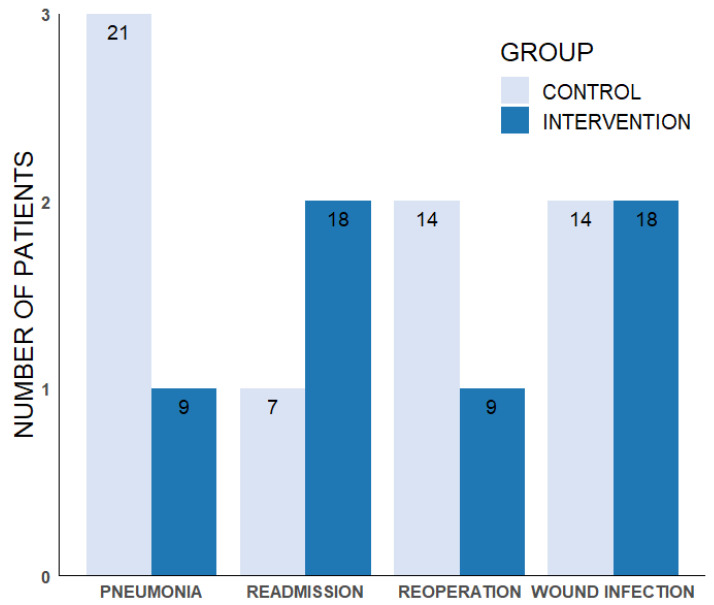
The number of patients who developed postoperative complications within 90 days after scoliosis surgery. Dark blue bars represent the intervention group, with a total of 11 patients. Light blue bars represent the control group, with a total of 14 patients. The numbers inside the bars show the percentage of patients with complications in each group.

Figure 2 illustrates the proportion of postoperative complications in the intervention group and the control group. There was no significant difference in the postoperative complication rates between the intervention and control groups. The median length of stay in hospital in the intervention group was 7 days (5–16 days) and 7 days (2–14 days) in the control group. Furthermore, two out of 11 patients (18%) in the intervention group compared to one out of 14 patients (7%) in the control group (Figure 2) were readmitted to hospital.

The data on the change in nutritional status, macronutrient and micronutrient dietary composition, and energy needs between the three study visits are presented in Supplementary Table S3. The weight, WAZ, triceps skinfold thickness, and mid-upper arm fat area in the intervention group did not significantly change between the three study visits (*p* = 0.9, *p* = 0.06, *p* = 0.7, *p* = 0.7). The estimated energy needs and the measured REE were not significantly different in the intervention group between all the three visits (*p* = 0.1). The nutritional blood sample results for the three visits in the intervention group are presented in Table S4. There were no significant differences in the fat percentage, fat mass, muscle mass, and bone mineral content, in kg/cm^2^, measured by the DXA scans, between the three visits in the intervention group (*p* = 0.89, *p* = 0.77, *p* = 0.95, *p* = 0.06). None of the patients were treated with Zoledronic acid, see Table S5.

#### 3.3.3. Quality of Life Assessment in the Intervention Group

The median total QOL score in the NMD patients (self-reported) during the study period was 69 points (visit 1), 63 points (visit 2), and 61 points (visit 3). The parents reported a lower QOL for all the categories than the patients (Figure S1). The median total QOL score in the parent-reported CP module was 19.3 points (visit 1), 15.7 points (visit 2), and 20.0 points (visit 3) (Figure S1). The parents reported that the children with CP had better balance, movement, and communication postoperatively, but were more fatigued and had more pain after the surgery compared to visit 1.

## 4. Discussion

In this single-site study, the proposed perioperative care protocol was implemented as planned, with a high level of compliance across all the study procedures. Our findings were as follows: (a) a lower rate of postoperative pneumonia was observed in the intervention group compared to the control group, although statistical significance was not reached, (b) 82% of the patients in the intervention group needed respiratory optimization before surgery, and (c) 73% of the patients in the intervention group needed nutritional optimization before surgery.

There was no statistical difference in the primary outcome, namely the pneumonia rate, between the intervention and control groups. The sample size is most likely too small to detect differences attributable to preoperative practices. Such a small sample reduces the study’s statistical power and its ability to identify true differences or effects. Consequently, the non-statistically significant difference in our primary outcome may represent a Type II error, resulting in a false negative finding. Furthermore, since the control group received standard care within the same period as the intervention group, there is a risk that some of the optimized interventions, such as early extubation criteria, were used on the control group. Tøndevold et al. introduced an enhanced recovery after surgery (ERAS) protocol in children with idiopathic scoliosis [21]. They found a positive collateral effect on patients with neuromuscular scoliosis undergoing surgery, even though the protocol was targeted at idiopathic scoliosis patients [22]. A recent study implemented the ERAS protocol in pediatric neuromuscular scoliosis patients. The researchers found a decrease in the length of stay in hospital without an increase in complications or readmissions in the group of patients who were subject to the ERAS protocol postoperatively [23]. Furthermore, an accelerated postoperative discharge pathway has been developed for non-ambulant patients with CP as well. This pathway has been shown to reduce the patient’s length of stay in hospital and pulmonary postoperative complications [24]. In accordance with this, we found that the preoperative protocol reduced the median length of stay in hospital with 4 days (7 days (range, 10–15) compared to 11 days (range, 10–15)) [9] compared to our retrospective study.

Another surgical site has described the optimization package they provide to patients with neuromuscular scoliosis on a pre-, intra-, and postoperative basis [25]. They perform nutritional and pulmonary assessments only one month before surgery, compared to three months in our study. Like our study, they used a multidisciplinary team approach. They conclude that an interdisciplinary team approach may reduce the risk of complications and may subsequently improve patient outcomes [25]. Antolovich et al. have also outlined the perioperative care pathway they have developed for children with neuromuscular scoliosis, aiming to guide decision-making and enhance patient health prior to surgery [26]. Both surgical sites assess preoperative lung function and nutritional status, with plans to optimize these factors and/or decide whether surgery will benefit the patient [25,26]. The studies did not statistically assess the effect of the optimization pathways. In line with this, another study has also found that the use of a multidisciplinary approach with standardized measures significantly reduced surgical site infections in pediatric scoliosis surgery [27]. These studies highlight the need for a multidisciplinary approach in regard to the treatment of this patient group [25,26,27].

Many of the patients in our intervention group had regular respiratory check-ups at RCE before their inclusion in the study. However, 82% of the patients received new respiratory recommendations at the study visit. Furthermore, three patients with CP who had never been seen at RCE were assessed to be in need of preventive respiratory devices after their visit. A study by Al-iede et al. found that preoperative NIV treatment reduced pulmonary complications in children with neuromuscular scoliosis and severely compromised lung function [28]. There are no specific guidelines regarding the referral of patients with CP to RCE at our hospital. The results of this study highlight the benefits and the importance of respiratory assessments of patients with CP.

In our previously published retrospective study, we found that 32% of the patients were malnourished [9]. In line with this, many of the patients in this prospective study were at nutritional risk and needed nutritional optimization before surgery. We did not observe a significant change in the weight of the patients in the intervention group during the optimization period. However, with the optimized protocol the patients managed to stabilize their weight or did not lose weight before surgery. This may indicate that the care protocol had an effect, as the aim in most of the patients preoperatively was for them to gain weight or stabilize their weight. However, weight gain has also been seen preoperatively in a retrospective study involving children with CP, irrespective of whether a nutritional consultation was conducted within one year before surgery or not [29]. Furthermore, the nutritional status and performing nutritional optimization in children with neuromuscular scoliosis are complex and challenging. Common patient complaints of nausea, reduced gastric emptying, and constipation present a challenge to energy optimization when patients are fed by using a feeding tube. Thus, a more extended optimization period with close contact from a dietitian may be warranted.

The strengths of the study are the extensive multidisciplinary care protocol used and the long preoperative optimization period compared to other surgical sites [25]. We consider the following as study limitations. The study was a single-center study, limiting the generalizability of our findings. Our sample size is small and the small sample size probably lacks sufficient power to detect differences between the groups. The limitation increases the likelihood of Type II errors, where true effects are overlooked. Furthermore, a small sample size can lead to increased variability in the results, making it difficult to draw reliable conclusions. A limited number of children with neuromuscular scoliosis with eligible diagnoses undergo scoliosis surgery at our surgical site and we were challenged by the COVID-19 pandemic in regard to recruitment. To further evaluate the effect of the optimized care protocol and validate our findings, future studies should focus on implementing the perioperative care protocol in a multicenter setting and extending the inclusion period to enroll a larger patient population. Furthermore, it would be interesting in future studies to investigate postoperative pain management and pain relief in the patient groups. This is complicated because comorbidities and the duration of surgery, which are common factors that impact neuromuscular scoliosis patients, are independent risk factors of poor analgesic effects postoperatively [30]. Even though we assume that the control group was randomly recruited, the control group differed from the intervention group because the patients were older and the distribution of the diagnoses was different, with more patients with CP in the control group. This is probably due to the patients with CP being monitored by pediatricians at other hospitals as well, while the study site has treatment responsibility for NMD patients. Thus, recruiting patients with CP with enough time for optimization before surgery was difficult. Moreover, there is a lack of reported patient data in regard to the control group, making it difficult to compare all the variables applicable to the intervention group with those in the control group. Lastly, we do not know whether the proposals regarding respiratory optimization implemented by the pulmonary specialists at RCE were followed by the families and the children.

## 5. Conclusions

The multidisciplinary protocol for the perioperative optimization of a high-risk patient group of children with neuromuscular scoliosis was feasible, with a high compliance rate. We found indications of a tendency toward lower pneumonia rates, a benefit of early extubation and preoperative referrals to a respiratory unit and a dietitian. Based on the findings in this study and the improvements compared to the findings in our retrospective study [9], we recommend the use of an optimized care protocol before neuromuscular scoliosis surgery, with special emphasis on the following aspects: respiratory and nutritional assessments before surgery, glucose infusion during fasting and surgery, and extubation in the OR or within 24 h after surgery.

## Figures and Tables

**Table 1:** Individual respiratory optimization recommended by the pulmonary specialists at the respiratory centre.

ID	Diagnosis	Respiratory Assistance Already in Use	Respiratory Optimization	Treatment for Nightly Hypoventilation
1	CP *	Peep mask, saline nebulization	Intermittent CPAP, MI-E	No
2	DMD	None	Intermittent CPAP, MI-E	No
3	SMA	None	Intermittent CPAP, MI-E	No
4	CP	Nightly CPAP, MI-E	None	Yes
5	CP *	None	Intermittent CPAP	No
6	SMA	Intermittent CPAP, MI-E (if needed)	MI-E (permanent)	No
7	MDC1A	NIV, intermittent CPAP	MI-E	Yes
8	SMA	Intermittent CPAP, MI-E, saline nebulization	None	Yes
9	CP	Intermittent CPAP, saline nebulization	MI-E, mouth suction	No
10	SMA II	Intermittent CPAP, MI-E (if needed)	Intermittent CPAP, MI-E (permanent)	No
11	CP	Intermittent CPAP, saline nebulization	NIV	No
14	CP *	PEEP mask	Intermittent CPAP	No

* Patients referred to the respiratory centre after study inclusion. Abbreviations: CP = cerebral palsy, CPAP = continuous positive airway pressure, DMD = Duchenne muscular dystrophy, MDC1A = merosin-deficient congenital muscular dystrophy, MI-E = mechanical insufflation-exsufflation device, NIV = non-invasive ventilation, PEEP = positive end-expiratory pressure.

**Table 2:** Demographics and intraoperative variables in the intervention and the control group.

Demographics		Intervention Group (*n* = 11)	Control Group (*n* = 14)	*p*-Value
Age		12 (8–17)	15 (11–17)	0.02 *
Sex (M/F)		8/3	8/6	0.3
Diagnosis	CP	5 (45.5%)	10 (71.4%)	0.5
	SMA	4 (36.4%)	2 (14.3%)	-
	DMD	1 (9.0%)	1 (7.1%)	-
	MDC1A	1 (9.0%)	1 (7.1%)	-
Non-ambulant		11 (100%)	13 (93%)	1.0
Weight (kg)		35.25 (18.1–68.0)	33.9 (20.2–71.0)	0.9
WAZ		−1.3 (−8.9–2.2)	−3.3 (−6.9–1.3)	0.1
Nutritional status	Severely underweight	4 (36.4%)	7 (50.0%)	0.4
	Underweight	1 (9.0%)	3 (21.4%)	-
	Overweight	1 (9.0%)	0	-
	Normal	5 (45.5%)	4 (28.6%)	-
Respiratory assistance, *n*	None	2	4	-
	PEEP mask	2	-	-
	MI-E	5	5	-
	Intermittent CPAP	7	5	-
	Nightly auto-CPAP	1	1	-
	NIV	2	3	-
Comorbidities	Psychomotor impairment	5 (45.0%)	10 (71.4%)	
	Intellectual disabilities	5 (45.0%)	11 (78.6%)	
	Epilepsy	4 (36.4%)	10 (71.4%)	
	Ventriculoperitoneal shunt	1 (9.0%)	1 (7.1%)	
	Cardiomyopathy	1 (9.0%)	0	
	Coagulation deficiency	1 (9.0%)	0	
	Immune deficiency	0	2 (14.3%)	
	Asthma	0	1 (7.1%)	
History of fractures		2 (18.2%)	n.a.	-
Low BMD total body		3 (27.3%)	3 *	
Low BMD lumbar spine		5 (45.5%)	n.a.	-
Number of days of optimization		133 (67–562)	n.a.	-
Preoperative Cobb angle		90 (35–122)	100 (65–140)	0.4
**Intraoperative variables**				
Duration of surgery (minutes)		248 (114–319)	241 (88–380)	0.3
Complications during surgery, number (%)		1 (9.0%)	1 (7.1%)	-
Blood loss (mL)		1000 (100–3300)	788 (50–1500)	0.4

Continuous variables are given as the median with a range. Categorical variables are given as numbers with percentages. * DXA scan was only performed for three of the controls. Abbreviations: BMD = bone mineral density, CP = cerebral palsy, CPAP = continuous positive airway pressure machine, DMD = Duchenne muscular dystrophy, F = female, MDC1A = merosin-deficient congenital muscular dystrophy, MI-E = mechanical insufflation-exsufflation device, mL = milliliters, n = numbers, n.a. = not available, NIV = non-invasive mechanical ventilation, PEEP = positive end-expiratory pressure, SMA = spinal muscular atrophy.

## Data Availability

The data presented in this study are available on request from the corresponding author due to privacy and ethical concerns.

## Supplementary Materials

The following supporting information can be downloaded at: https://www.mdpi.com/article/10.3390/jcm13247848/s1, Protocol S1 [20,31], Table S1: Nutritional optimization [20,31], Table S2: Individual nutritional recommendations for nutritional optimization, Table S3: Nutritional status, macronutrient and micronutrient diet composition, and energy needs between the three study visits in the intervention group, Table S4: Blood sample results for the intervention group, Table S5: The results of the DXA scans, Figure S1: Quality of life questionnaire results for the intervention group.
