# Supplementary material for: A Prospective Study on the Feasibility and Effect of an Optimized Perioperative Care Protocol in Pediatric Neuromuscular Scoliosis Surgery
Source: J Clin Med. 2024 Dec 23;13(24):7848. doi: 10.3390/jcm13247848 (PMC11676504; doi:10.3390/jcm13247848)
Supplement: Supplementary file 1 [file jcm-13-07848-s001.zip › Figure S1.pdf]

Figure S1 - Life Quality Questionnaires results in the intervention group

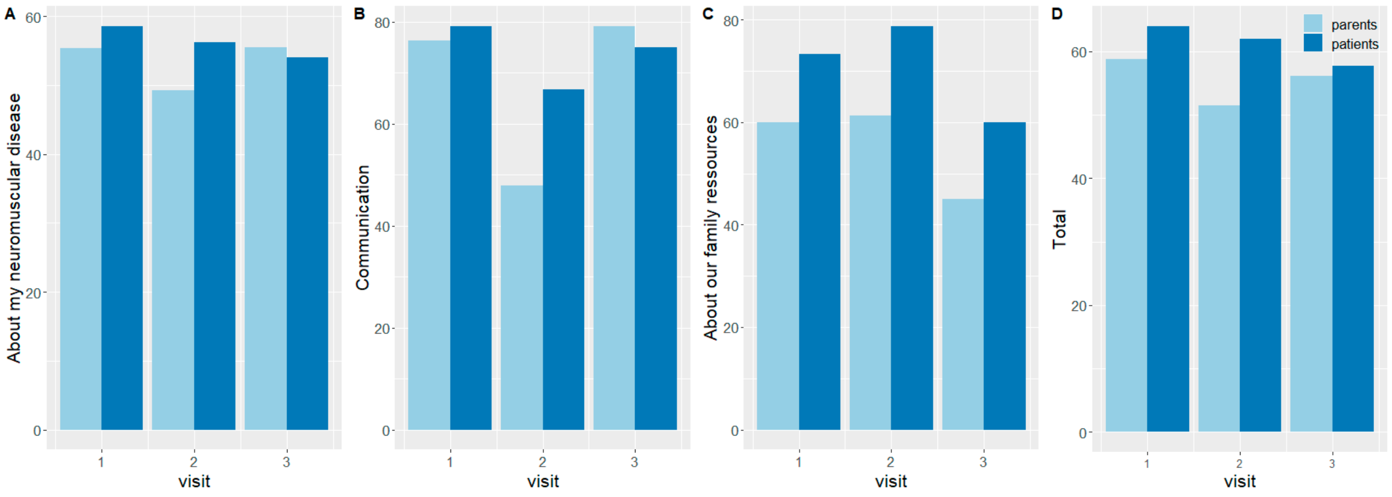

Quality of life in children with neuromuscular disorders. The mean score in the three domains and the total score in the self-reported and parent-reported neuromuscular disorder life quality module at visits 1, 2 and 3.

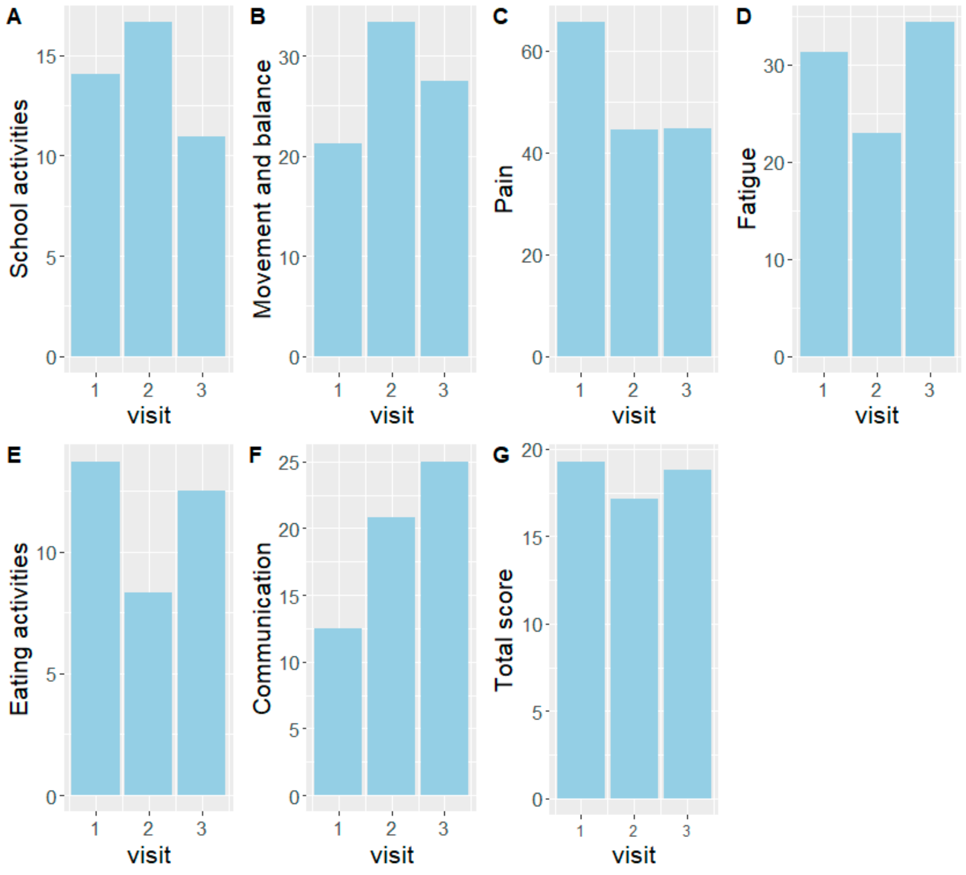

Quality of life in children with cerebral palsy. The mean score in the six domains and the total score in the parent-reported cerebral palsy life quality module at visits 1, 2, and 3.
