# Supplementary material for: A Prospective Study on the Feasibility and Effect of an Optimized Perioperative Care Protocol in Pediatric Neuromuscular Scoliosis Surgery
Source: J Clin Med. 2024 Dec 23;13(24):7848. doi: 10.3390/jcm13247848 (PMC11676504; doi:10.3390/jcm13247848)
Supplement: Supplementary file 1 [file jcm-13-07848-s001.zip › Protocol S1.pdf]

Protocol S1 – Detailed description of the assessments at the study visits and the optimised perioperative protocol

Assessments

Nutritional screening

The dietitian performed a nutritional screening of the patients. The parents were asked to complete an internet-based three-day food record (MadLog Classic Aps, Kolding, Denmark) to identify the patient's food intake and calculate the macro and micro-nutrient intake in the diet. The average of the three days was used to assess whether the patient had an adequate intake of macro- and micronutrients. The dietitian calculated the estimated energy need recommended by the Nordic Nutrition recommendations using calculations given by Henry[1]. The weight was measured on-site, if a lift was available, or reported by the parents. Severe underweight and underweight were defined as weight for age z-score (WAZ) ≤ -3 SD and WAZ ≥ -3 & ≤ - 2 SD. Overweight was defined as WAZ ≥ 2 SD. The clinical dietitian used the European Society for Paediatric Gastroenterology Hepatology and Nutrition (ESPGHAN) guidelines to assess whether the child was at nutritional risk or not[2]. The patient was at nutritional risk if one or more of the following red flag warning signs were present:
1) Physical signs of undernutrition, such as decubitus, skin problems, and poor peripheral circulation
2) WAZ < -2 SD
3) Triceps skinfold thickness < 10th centile for age and sex
4) Mid-upper arm fat or muscle area < 10th percentile
5) Faltering weight and/or failure to thrive

Indirect calorimetry test

The patients underwent an indirect calorimetry test to calculate their daily energy expenditure (REE) in kcal by measuring oxygen consumption (VO_2) and carbon dioxide production (VCO_2). A mask was connected to a metabolic meter, Quark CPET, Cosmed, Italy and was used to measure the VO_2 in inhaled air and VCO_2 in exhaled air. We aimed to measure 30 minutes of rest without the patient being asleep or stimulated with, for example, tablets. We stopped the measurement if the child became sad or uncomfortable.

Respiratory assessment at the Respiratory Center East (RCE)

Protocol S1 – Detailed description of the assessments at the study visits and the optimised perioperative protocol

RCE is one of three highly specialised respiratory centers in Denmark that treats children and adults with chronic respiratory insufficiency due to among others neuromuscular disorders, such as muscular dystrophies. The treatments include preventive secrete management as well as mechanical ventilation. According to the optimised perioperative protocol, patients already followed by the RCE were invited for a visit to assess if further optimisation of the respiratory treatment was needed prior to surgery. Patients not followed at the RCE were referred to a visit at RCE if they had a history of respiratory challenges and/or if their forced vital capacity (FVC) measured by spirometry was < 70% of expected value or peak cough flow < 270 l/min. It was up to the individual pulmonary specialist to decide if preventive secrete management treatment including intermittent continuous positive airway pressure (CPAP) therapy or mechanical insufflation-exsufflation (MI-E) therapy or isotonic saline nebulization should be started or intensified, if a test for a sleep-related breathing disorder should be performed and if a continuous positive airway pressure treatment for nightly use should be started or optimised.

Dual-energy X-ray absorptiometry (DXA) scans

The bone mineral density of the lumbar spine and the total body were obtained from the total body scanner, DXA scan (Lunar Prodigy Pro, GE Healthcare). Age- and sex-specific z-scores were calculated using the Lunar software. A z-score < -1.9 was defined as a low bone mineral density (BMD). The total fat mass in kg, percentage of fat and fat-free mass were also obtained from the DXA scan.

Blood samples

Blood samples were analysed for kidney biomarkers (potassium, sodium, creatinine, urea), liver biomarkers (alanine aminotransferase, aspartate aminotransferase, alkaline phosphatase, bilirubin, INR), bone metabolism (parathyroid hormone, calcium, phosphate), lipid profile (triglycerides, cholesterol, cholesterol-LDL, cholesterol-HDL), and vitamins and minerals (magnesium, zinc, 25-OH-vitamin D, vitamin A, vitamin E).

Life quality questionnaires (QOL)

The paediatric life quality inventory (PedsQL) CP parent module and neuromuscular disease (NMD) module for patients and parents were used to assess self-reported and parent-reported QOL. The

PedsQL includes questions in different domains depending on the CP or NMD module. The NMD module consists of three domains: “About my neuromuscular disease”, “Communication”, and “About our family resources”. The CP module consists of seven domains: “Daily activities, “School activities”, “Fatigue”, “Pain”, “Movement and balance”, “Eating activities”, and “Speech and communication”. The scale was a Likert scale from 0 (Never) to 4 (Almost always). The recall period was four weeks, and the scale ranged from 0 to 100. Higher scores are equivalent to better health-related quality of life (HRQOL) and fewer problems and symptoms. We aimed for both children and parents to complete the questionnaires. They completed the questionnaires on visits 1, 2 and 3.

Protocol S1 – Detailed description of the assessments at the study visits and the optimised perioperative protocol

Optimised perioperative care protocol

The optimised care protocol included the following:

(1) Preoperative optimisation

The dietitian determined if the patient needed nutritional optimisation before surgery based on the indirect calorimetry test results, blood samples, DXA scan, food records, and nutritional screening. If nutritional optimisation was needed, an individualised nutritional optimisation plan was made with regular follow-ups by telephone with the dietitian. The pulmonary specialists made individualised optimisation plans after the respiratory assessment at the RCE.

(2) Intraoperative optimisation

The patients were all treated with 5% glucose (Plasmalyte 50 mg/ml - Baxter AS) calculated according to weight intravenously (IV) from the start of surgery until sufficient intake could be covered by oral intake or with a gastrostomy tube.

(3) Postoperative optimisation

The postoperative optimisation included:

- New extubating criteria to ensure quick extubation if possible
- Nutritional assessment and optimisation by the clinical dietitian
- A consultation by a paediatrician on the first day of hospital admission

References

1. *Nordic Nutrition Recommendations 2012 : Integrating Nutrition and Physical Activity*. (Nordisk Ministerråd, 2014).
2. Romano, C. *et al.* European Society for Paediatric Gastroenterology, Hepatology and Nutrition Guidelines for the Evaluation and Treatment of Gastrointestinal and Nutritional Complications in Children With Neurological Impairment. *J. Pediatr. Gastroenterol. Nutr.* **65**, 242 (2017).
