# Supplementary material for: A Prospective Study on the Feasibility and Effect of an Optimized Perioperative Care Protocol in Pediatric Neuromuscular Scoliosis Surgery
Source: J Clin Med. 2024 Dec 23;13(24):7848. doi: 10.3390/jcm13247848 (PMC11676504; doi:10.3390/jcm13247848)
Supplement: Supplementary file 1 [file jcm-13-07848-s001.zip › Table S1_0612.pdf]

Table S1 – Nutritional optimization method

Table S1. Nutritional optimization

Assessment	Criteria	Optimization goals
**Nutritional screening**	One or more of the red flag warning signs for undernutrition	Increasing energy intake to gradually meet energy needs*
**Diet registration**	Energy intake < calculated or measured energy needs<br><br>and/or<br>Protein intake < recommended intake by NNR 2012<br><br>and/or<br>Micronutrient intake < recommended intake by NNR 2012	Increasing energy intake to gradually meet energy needs*<br><br>Increasing protein intake to meet requirements*<br><br>Increasing micronutrient intake according to recommendations*
**Blood sample**	Results < normal serum levels	Increasing micronutrient intake according to recommendations or national guidelines for e.g. vitamin D or Iron deficiency
**DXA scan**	BMD z score < 2	Increasing Calcium and vitamin D intake according to recommendations

Abbreviations: NNR = Nordic Nutrition Recommendations[1], BMD = bone mineral density.
*ESPGHAN recommends the use of dietary reference standards for typically developing children to estimate the caloric needs and protein and micronutrient requirements for children with neurological impairment[2].

References:

[1] Nordic Nutrition Recommendations 2012 : Integrating nutrition and physical activity. Nordisk Ministerråd; 2014.
[2] Romano C, van Wynckel M, Hulst J, Broekaert I, Bronsky J, Dall'Oglio L, et al. European Society for Paediatric Gastroenterology, Hepatology and Nutrition Guidelines for the Evaluation and Treatment of Gastrointestinal and Nutritional Complications in Children With Neurological Impairment. J Pediatr Gastroenterol Nutr 2017;65:242. https://doi.org/10.1097/MPG.0000000000001646.
