# Supplementary material for: A Prospective Study on the Feasibility and Effect of an Optimized Perioperative Care Protocol in Pediatric Neuromuscular Scoliosis Surgery
Source: J Clin Med. 2024 Dec 23;13(24):7848. doi: 10.3390/jcm13247848 (PMC11676504; doi:10.3390/jcm13247848)
Supplement: Supplementary file 1 [file jcm-13-07848-s001.zip › Table S2_0612.pdf]

Table S2 – Individual nutritional recommendations for nutritional optimisation

ID	Gastrostomy tube	Diet optimisation	Micronutrient optimisation
**1**	Yes	• Increase intake of 500 kJ<br>• Increase protein intake<br>• Increase amount of monounsaturated fat<br>• Increase carbohydrates	• Iron supplements (6.5 mg)<br>• Multivitamins and minerals (20 ml/day)<br>• Calcium supplements<br>• Vitamin D supplement (38 µg)
**2**	No	No*	• Increase intake of calcium supplements<br>• Increase intake of vitamin D supplements<br>• Reduce intake of magnesium supplements
**3**	No	• Reduce from 3 to 2 Resource Minimax**<br>• Reduce intake of protein<br>• Reduce intake of kJ	• Calcium supplement (1200 mg)<br>• Vitamin D supplement (11.8 µg)
**4**	Yes	• 10% increase in energy intake<br>• Add maltodextrin	• Zinc supplement (15 mg/day)
**5**	Yes	• Increase of Fresubin Original Fibre** by 10%	• Calcium supplements (400 mg)<br>• Vitamin D supplements (10 µg)
**6**	Yes	• Add 2 x 10 ml oil<br>• Two spoons with maltodextrin	No
**7**	Yes	• Peptamen Junior Advance **	• Multivitamin and minerals
**8**	Yes	No	No
**10**	No	No*	No
**11**	No	No	No
**14**	Yes	• Change from Nutridrink MF to Fortini MF**<br>• Reduce protein intake	• Vitamin D supplement (5 µg)

Individual nutritional recommendations of optimisation from the clinical dietitian on visit 1. *The patient was already in relevant nutritional optimization treatment to reduce weight. Abbreviations: kJ= kilojoule, **Food for Special Medical Purposes.
