# Supplementary material for: A Prospective Study on the Feasibility and Effect of an Optimized Perioperative Care Protocol in Pediatric Neuromuscular Scoliosis Surgery
Source: J Clin Med. 2024 Dec 23;13(24):7848. doi: 10.3390/jcm13247848 (PMC11676504; doi:10.3390/jcm13247848)
Supplement: Supplementary file 1 [file jcm-13-07848-s001.zip › Table S3_0612.pdf]

Table S3 –Nutritional status, macronutrient and micronutrient diet composition and energy needs between the three study visits in the intervention group

**Nutritional assessments (n=11)**		**Baseline (visit 1)**	**Preoperatively (visit 2)**	**Postoperatively (visit 3)**
WAZ		-1.3 (-8.9-2.2)	-2.3 (-10.1-2.2)	-1.5 (-9.3-1.9)
Weight		35.5 (18.1-68.0)	38.5 (17.6-61.0)	40.0 (19.3-60.8)
Weight change in percentage		-	1.7 (-15.4-20.0)	0 (-16.0-18.2)
Route	Full oral diet	4 (36.4%)	4 (36.4%)	4 (36.4%)
	PEG tube + oral	4 (36.4%)	2 (18.2%)	2 (18.2%)
	PEG tube	3 (27.3%)	5 (45.5%)	5 (45.5%)
Triceps skinfold thickness		1.5 (1.0-2.9)	1.4 (1.1-2.9)	1.3 (0.1-2.8)
Mid-upper arm fat area		15.3 (8.8-39.8)	13.9 (7.6-38.3)	15.4 (0.8-38.4)
Nutritional risk		6 (54.5%)	7 (63.6%)	6 (54.5%)
Swallowing difficulties		5 (45.5%)	5 (45.5%)	6 (54.5%)
Chewing difficulties		5 (45.5%)	6 (54.5%)	6 (54.5%)
Dietary supplements (yes/no)		9 (81.8%)	10 (90.9%)	11 (100%)
Estimated energy need kcal		1346 (1120-1792)	1300 (1120-1785)	1400 (1120-1905)
Measured energy needs kcal		1658 (1375-1839) (n=6)	1824 (1250-1956) (n=5)	1596 (1034-2196) (n=7)
**Diet registration – intake**		**N=11**	**N=7**	**N=6**
% intake of estimated energy need		93.8 (66.1-167.7)	98.0 (79.1-121.4)	102.7 (86.6-120.6)
*Fat %		33 (15-72)	36 (31-40)	35 (26-38)
*Protein %		15 (10-20)	12 (10-18)	16 (11-21)
*Carbohydrates %		49 (17-62)	50 (47-53)	50 (46-54)
Dietary fibre %		2.3 (0-4.3)	1.0 (0-4.3)	1.0 (0-1.3)
**Vitamin C		84.7 (2.0-335.0)	88.8 (39.3-193.3)	114.5 (36.0-299.0)
**Vitamin D		9.4 (0-19.3)	7.2 (0.4-20.9)	12.5 (0.8-20.9)
**Calcium		720 (482-1263)	1400	NA
