# Supplementary material for: A Prospective Study on the Feasibility and Effect of an Optimized Perioperative Care Protocol in Pediatric Neuromuscular Scoliosis Surgery
Source: J Clin Med. 2024 Dec 23;13(24):7848. doi: 10.3390/jcm13247848 (PMC11676504; doi:10.3390/jcm13247848)
Supplement: Supplementary file 1 [file jcm-13-07848-s001.zip › Table S4.pdf]

Table S4 - Blood sample results in the intervention group

Blood samples	Baseline	Preoperatively	Postoperatively
Albumin	42 (39-40)	38 (31-44)	38 (33-40)
Folic acid	34.0 (21.7-45.4)	31.9 (10.0-45.4)	33.4 (9.7-45.4)
Calcium	2.5 (2.3-2.6)	2.4 (2.3-2.5)	2.4 (2.3-2.6)
Zinc	10 (2-13)	10 (9-14)	10 (7-16)
Vitamin D	68 (48-87)	65 (24-103)	77 (57-115)
Magnesium	0.95 (0.88-1.03)	0.88 (0.75-0.94)	0.89 (0.80-0.93)
Phosphate	1.40 (1.10-1.69)	1.39 (1.15-1.73)	1.45 (1.10-3.10)
Vitamin E	25 (10-32)	24 (18-33)	29 (19-47)
Vitamin B12	520 (305-550)	503 (289-807)	420 (278-712)
PTH	3.5 (2.5-4.6)	4.2 (2.6-8.6)	3.15 (1.4-3.5)

Values are given as median with a range in parenthesis.
