# Supplementary material for: A Prospective Study on the Feasibility and Effect of an Optimized Perioperative Care Protocol in Pediatric Neuromuscular Scoliosis Surgery
Source: J Clin Med. 2024 Dec 23;13(24):7848. doi: 10.3390/jcm13247848 (PMC11676504; doi:10.3390/jcm13247848)
Supplement: Supplementary file 1 [file jcm-13-07848-s001.zip › Table S5_0612.pdf]

Table S5 - Results of the DXA scan

**DXA scan**	**Baseline (Intervention group) N=11**	**Baseline (Control group) N=3**	**Preoperatively (Visit 2)**	**Postoperatively (Visit 3)**
BMD z-score total body	-1.5 (-3.1-1.3)	-3.2 (-3.7-2.2)	-0.8 (-2.6-1.0)	0.0 (-2.6-1.8)
BMD (g/cm^3)	0.74 (0.64-0.99)	0.75 (0.67-1.52)	0.79 (0.66-1.0)	0.89 (0.83-1.2)
Fat mass (kg)	12.95 (4.9-37.2)	12.4 (11.2-42.4)	14.48 (5.37-34.65)	13.52 (5.46-33.0)
Fat mass %	36.7 (27.8-58.6)	43.6 (43.4-61.1)	36.4 (28.8-58.2)	40.3 (30.1-58.5)
Muscle mass (kg)	20.55 (12.15-30.75)	16.0 (14.6 – 27.0)	21.59 (12.03-28.82)	23.04 (12.39-28.88)

Values are given as median with range in parenthesis. Abbreviations: BMD= bone mineral density, DXA= dual energy x-ray absorptiometry scan
